# Long-Term Soft-Food Rearing in Young Mice Alters Brain Function and Mood-Related Behavior

**DOI:** 10.3390/nu15102397

**Published:** 2023-05-20

**Authors:** Masae Furukawa, Hirobumi Tada, Resmi Raju, Jingshu Wang, Haruna Yokoi, Mitsuyoshi Yamada, Yosuke Shikama, Kenji Matsushita

**Affiliations:** 1 Department of Oral Disease Research, Geroscience Research Center, National Center for Geriatrics and Gerontology, Obu 474-8511, Japan; karuvachattu@gmail.com (R.R.); wjsh@ncgg.go.jp (J.W.); haruna.yokoi@ncgg.go.jp (H.Y.); mitsuyos@dpc.agu.ac.jp (M.Y.); shikama@ncgg.go.jp (Y.S.); 2 Department of Nutrition, Faculty of Wellness, Shigakkan University, Obu 474-8651, Japan; tada@sgk.ac.jp; 3 Department of Integrative Physiology, Geroscience Research Center, National Center for Geriatrics and Gerontology, Obu 474-8511, Japan; 4 Department of Operative Dentistry, School of Dentistry, Aichi Gakuin University, Nagoya 464-8651, Japan

**Keywords:** hippocampus, aggression, soft food, serotonin

## Abstract

The relationship between caloric and nutrient intake and overall health has been extensively studied. However, little research has focused on the impact of the hardness of staple foods on health. In this study, we investigated the effects of a soft diet on brain function and behavior in mice from an early age. Mice fed a soft diet for six months exhibited increased body weight and total cholesterol levels, along with impaired cognitive and motor function, heightened nocturnal activity, and increased aggression. Interestingly, when these mice were switched back to a solid diet for three months, their weight gain ceased, total cholesterol levels stabilized, cognitive function improved, and aggression decreased, while their nocturnal activity remained high. These findings suggest that long-term consumption of a soft diet during early development can influence various behaviors associated with anxiety and mood regulation, including weight gain, cognitive decline, impaired motor coordination, increased nocturnal activity, and heightened aggression. Therefore, the hardness of food can impact brain function, mental well-being, and motor skills during the developmental stage. Early consumption of hard foods may be crucial for promoting and maintaining healthy brain function.

## 1. Introduction

Diet is an important contributor to human health, and public health agencies issue guidelines to promote healthy food choices. For example, based on scientific evidence, the U.S. Department of Health and Human Services and U.S. Department of Agriculture have collaborated to publish dietary guidelines that suggest ideal dietary patterns for all stages of life. Limiting the intake of total calories, trans fats, and salt has been shown to be important in preventing noncommunicable diseases, such as diabetes, heart disease, stroke, and cancer [1]. Additionally, the benefits of vitamins, polyphenols, and other compounds in foods for disease prevention have been widely reported [2,3]. Conversely, there is insufficient evidence regarding the effects of food hardness on health.

Food hardness may influence the number of chews and chewing time [4,5]. The importance of mastication has been reaffirmed in recent years, as it has been suggested that mastication has numerous positive effects on the entire body [6,7]. Oral stimulation (i.e., the time spent tasting and chewing food) increases diet-induced heat production (DIT) and benefits health [8]. Moreover, chewing improves cognitive function and exerts a relaxation effect [9].

Reduced mastication inhibits jaw development, reduces oral function, and decreases saliva outflow, causing dental caries and periodontal disease [10]. Additionally, the absence of chewing can decelerate digestion and lead to various health problems, such as obesity, and increased incidence of lifestyle-related diseases [11]. Previous studies have reported outcomes, such as reduced lifespan [12], premature aging [13], and increased reactive oxygen species levels in the brain owing to powder-based diets [14]. Mastication also plays an important role in cognitive function [15,16]. In a mouse model, tooth loss was associated with a reduction in neuron number and the dysregulation of aging-related genes in neurons in the hippocampus and hypothalamus, along with a decline in learning, cognition, and motor functions [17]. These findings suggest that masticatory stimulation plays a pivotal role in cognitive function, as it provides the necessary neural input for maintaining neuronal integrity and supporting optimal brain functions [18]. Interestingly, chewing stimulation affects children more than the elderly [19].

Jelly-like foods, such as functional nutritional foods and nutritional supplements, have become easily available. Despite differences in the oral processing time between young and elderly people [20], nutritional supplementation is possible without chewing. Thus, the importance of food education from early childhood has been demonstrated in recent years [21]. In general, the effects of food form on cognitive function have already been examined [22,23,24]. However, almost no studies on the impact that changes in diet form may have on physiological or behavioral variables have been performed, including their potential impact on daily activity and levels of aggression.

Recently, mental health problems in children, represented by “losing one’s temper”, have been associated with serious incidents and are of great social concern [25,26]. Various lifestyle factors—such as TV viewing, stress, socioeconomic variables, and dietary factors—have been associated with adolescent aggression [27,28]. In adolescent girls, dietary patterns have been reported to be associated with depression and aggression [29]. The consumption of “junk food” (high energy foods with low nutritional value) in adolescents and children may increase the risk of mental illness and aggressive behavior [30]. Overeating, picky eating, and missed meals affect physical health, thus, diet affects mental health [31].

In this study, we observed changes in activity and aggression, as well as in cognitive and motor functions, when young mice were fed a soft diet for an extended period of time. We then analyzed the dynamics of brain molecules related to these functions and behaviors.

## 2. Materials and Methods

### 2.1. Animals

We used 3-week-old male C57BL/6N mice (*n* = 65) housed in an air-conditioned clean room at the National Center for Geriatric Research and Gerontology (NCGG). All experiments were approved by the Animal Welfare Committee of the NCGG (Approval No. 3-33). Mice were kept under a 12-h light/dark cycle, with the lights turned off from 19:00 to 07:00.

Mice were randomly divided into three groups as follows: Control (C, solid diet; *n* = 14), soft-fed (S, soft diet; *n* = 31), and soft-fed and solid modified diet (SH, soft-fed and solid diet; *n* = 20) groups. All mice were fed the CLEA Rodent Diet CE-2 (CLEA Japan Inc., Tokyo, Japan), a standard, GLP-compliant rodent diet consisting mainly of vegetable protein (soybean meal) with a proper balance of animal protein. The C group was fed standard CE-2 laboratory pellets and water ad libitum, while the S group was regularly fed the CE-2 powder mixed with 60% water. The SH group was reintroduced to a solid diet after 3 months of soft-fed rearing. The diet was changed to a solid diet, as in the C group, for 3 months. All groups could feed and drink water ad libitum (Figure S1).

Subsequently, we evaluated the feed and water intake of each group after 6 months of rearing (for the S group, the amount of powder consumed without water was measured). Each mouse was subjected to behavioral experiments and euthanized after 6 months by intraperitoneal injection of medetomidine hydrochloride diluted in saline to 0.9 mg/kg + 12 mg/kg midazolam, or 90 mg/kg alfaxalone + 15 mg/kg butorphanol.

All experiments were conducted in accordance with the Guide for the Care and Use of Laboratory Animals published by the National Institutes of Health (NIH Publication, eighth edition, 2011). The study was conducted in accordance with the Animal Research: Reporting of In Vivo Experiments guidelines and in compliance with the ARRIVE guidelines.

### 2.2. Body Weight and General Health

Following 7 days of habituation, all mice were weighed on a laboratory scale accurate to 0.01 g and inspected for signs of ill health, including wounds and poor body condition. Body weight was measured every week. Furthermore, to confirm the nutritional status, we extracted RNA from the hippocampus 6 months after rearing, and *Glut1* expression was examined via real-time polymerase chain reaction (PCR).

### 2.3. Blood Tests

#### 2.3.1. Preparation of Blood Serum

In the present study, the mice were sacrificed between 10:00 and 12:00 for blood collection, as described above. They were rapidly decapitated, and the trunk blood (approximately 1 mL) was collected into centrifugal tubes (CJ-2AS; Terumo, Tokyo, Japan) and centrifuged (3000× *g*, 10 min, 15 °C) to separate the serum. The collected serum was divided into three parts for: (i) Biochemical testing, (ii) corticosterone assay, and (iii) serotonin assay. The serum samples were stored at −80 °C until analysis.

#### 2.3.2. Serum Biochemistry Test

The physiological (albumin, glucose, T-CHO) and corticosterone testing of serum was contracted to the Nagahama Biological Science Laboratory (Nagahama, Shiga, Japan) of Oriental Yeast Co., LTD.

#### 2.3.3. Serum 5-Hydroxytryptamine (5-HT) Test

We examined serotonin levels in the serum using a serotonin enzyme-linked immunosorbent assay kit (Enzo Life Sciences Inc., Farmingdale, NY, USA) according to the instruction manual.

### 2.4. Behavioral Testing

#### 2.4.1. Y-Maze Test

We used the Y-maze test to evaluate the mechanisms by which soft-food rearing affected the learning and memory performance of mice (*n* = 5–6, per group). First, we assessed spontaneous alternation behavior in a gray, acrylic, Y-shaped maze that comprised three arms (60 cm long × 60 cm wide × 25 cm deep; YM-3002; O’Hara and Co., Ltd., Tokyo, Japan) projecting from each side of a central equilateral triangle. The Y-maze test was performed as specified by Wahl et al. [32], and the learning rate was based on the number of sessions required to meet the specified criterion.

A mouse was placed in one arm (No. 1), where it received the following three options as its first choice: Staying in arm one, moving into arm two, or moving into arm three. An alternation was considered correct if the mouse visited a different arm and did not return to the two previously visited arms. We calculated the ratio of correct alternations to the number of visits during a 10-min observation period as the frequency of alternation. A frequency >50% indicated spontaneous alternation. The test was repeated, and the number of correct responses was used as a measure of memory.

The percentage of alternation was the dependent variable [32], which was calculated as follows:

(1) $$\left(\frac{\mathrm{the}\;\mathrm{number}\;\mathrm{of}\;\mathrm{alternations}\;}{\mathrm{total}\;\mathrm{entries}\;-2}\right)\times 100$$

#### 2.4.2. Motor Skill Learning Test

We used a rotarod machine with automatic timers and falling sensors (MK-660D; Muromachi Kikai Co., Ltd., Tokyo, Japan) (*n* = 5–6, per group). We referred to the standard operating procedure of the RIKEN BioResource Center (Saitama, Japan). Each mouse was placed on a 9-cm (diameter) drum. The drum surface was covered with hard chloroethylene, which prevented surface gripping. The unit was set to accelerate from 4 to 40 rpm in 300 s. Following the drop, the animal was allowed to rest for 20 min and was subsequently returned to the drum (maximum twice in one session). To evaluate long-term memory, we repeated the test once daily for two consecutive days. The daily latency on the rod until the mouse fell was automatically recorded.

#### 2.4.3. Measurement of Aggressive Biting Behavior (ABB)

An aggression response meter was purchased from Muromachi Kikai Co., Ltd. (Tokyo, Japan) and used to measure the intensity and frequency of ABB toward an inanimate object [33]. Briefly, the subject mouse was placed in the cylinder on the top of the aggression response meter. This was followed by ABB measurement over two sessions (*n* = 6–7 per group). The apparatus contains computer-controlled sticks that stimulate the mouse through touch, thus inducing irritation and anger. When the mouse bites the sticks, a load sensor attached to the sticks dynamically detects ABB. Metal rods with a dome-shaped head were automatically controlled to generate an up-and-down motion 30 times to touch the hind limb of the subject mouse in the first session. The rods were positioned in front of mice in the second session. The load sensor connected to the rods measured the ABB intensity (the average intensity of one bite) and frequency (the total number of bites within 30 presentations). The ABB intensity is expressed in numerical values as an integral of the biting force within 1 s of presentation of the rods (milliNewton × second: mNs).

#### 2.4.4. Twenty-Four-Hour Locomotor Activity

Locomotor activity was measured as specified by Yamasaki et al. [34]. The mice were housed under a 12-h light/dark cycle in the rearing cage and subsequently reared individually (*n* = 1 per cage, *n* = 8 in total). Food and water were provided *ad libitum*. We determined the amount of exercise in the breeding cage using an infrared photobeam sensor (Time HC8 Single; O’ Hara and Co., Ltd., Somerdale, NJ, USA) and calculated the total distance moved by the mice per hour.

### 2.5. Real-Time PCR

To elucidate the molecular changes occurring in the brain following soft-food rearing, we evaluated the gene expression related to memory and cognitive functions in the hippocampus using real-time PCR and immunostaining (*n* = 5–6, per group). To assess the expression of aggression-related genes in the hippocampus, we examined T-cadherin (*Cdh13*) and monoamine oxidase A (*Maoa*), two familiar aggression genes [35]. Briefly, total RNA was isolated from the hippocampus using the NucleoSpin RNA kit (cat. U0955C; Takara Bio Inc., Shiga, Japan), according to the manufacturer’s instructions. The total RNA concentration was adjusted to 100 ng/μL using a NanoDrop^TM^ 2000 spectrophotometer (Thermo Fisher Scientific, Waltham, MA, USA). We performed first-strand cDNA synthesis using a ReverTra Ace-α Kit (Toyobo Co., Ltd., Osaka, Japan), and PCR was performed using the FastStart Essential DNA Green Master (Roche, Mannheim, Germany) and the LightCycler 96 System (Roche, Mannheim, Germany) according to the manufacturer’s protocol.

Table 1 summarizes the primer sequences. The target gene expression was normalized to that of the housekeeping gene β-actin, and the results are presented for each sample relative to the control. These experiments were performed in triplicate for each condition. Values are presented as the fold change between samples using the 2^–ΔΔCt^ method [36].

### 2.6. Immunohistochemistry

We immunohistochemically analyzed at least three mice brains per group, and a minimum of 10 sections were quantified per mouse. Brains were excised from the experimental mice and immersed in phosphate-buffered saline (PBS) for 24 h. Serial coronal sections (50 μm) obtained at the level of the bregma (1.5–2 mm posterior ± 1.2–1.3 mm mediolateral) were immunohistochemically processed using a VT1200S microtome (Leica Microsystems, Wetzlar, Germany). Nonspecific binding was blocked for 1 h using PBS containing 1% of Triton X-100 and 10% of normal donkey serum (S30-100 mL; Merck Millipore, Burlington, MA, USA). The brain slices were incubated overnight (with shaking) at 4 °C with the corresponding primary antibodies diluted in PBS, i.e., anti-c-Fos (1:1000, MCA-2H2; EnCor Biotechnology Inc., San Francisco, CA, USA), anti-NeuN (1:1000; ab104224; Abcam, Cambridge, UK); anti-brain-derived neurotrophic factor (BDNF) (1:500, ab205067; Abcam), anti-MAOA (1:500, ab126751; Abcam), anti-T-cadherin (1:50, #PA5-20158; Invitrogen, Waltham, MA, USA), anti-serotonin (1:400, ab66047; Abcam), anti-5-hydroxy-tryptamine (5-HT) subtype 2A (5-HT2A; 1:300, ab66049; Abcam), anti-5-HT subtype 2C (5-HT2C; 1:100, ab137529; Abcam), anti-tryptophan hydroxylase 2 (TPH2; 1:500, NB100-74555; Novus Biologicals, LLC, Centennial, CO, USA) and anti-glutamate receptor 1 (α-amino-3-hydroxy-5-methyl-4-isoxazole propionic acid [AMPA] subtype (GluA1); 1:100, ab31232; Abcam).

Following incubation with the primary antibody, the tissue slices were washed thrice with PBS and subsequently incubated for 1 h at 25 °C with subtype-specific fluorescent secondary antibodies, i.e., goat anti-rabbit immunoglobulin G (IgG; H + L, Alexa Fluor^®^ 488, 1:200, ab150077; Abcam), donkey anti-goat IgG (H + L, Alexa Fluor 594, 1:200, ab150132; Abcam) and donkey anti-mouse IgG (H + L, Alexa Fluor 555, 1:200, ab150110; Abcam), with shaking. Sections were observed using Keyence BZ-X800 (KEYENCE Co., Osaka, Japan). We calculated the number of positive cells in the hippocampal cornu ammonis using ImageJ software v1.52a (NIH, Baltimore, MA, USA), and three individuals performed a quantitative analysis of each immunolabeled area.

### 2.7. Statistical Analyses

All values are presented as mean ± standard error of the mean. To assess statistical differences between the groups, we conducted a one-way ANOVA followed by post hoc analyses using Tukey’s test. Statistical significance was set at *p* < 0.05. All statistical analyses were performed using EZR [37], a package for R software (Saitama, Japan), and a modified version of the R commander that incorporates commonly used statistical functions in biostatistics.

## 3. Results

### 3.1. Changes in Body Weight and Serum Composition Due to Soft Feeding

**Figure 1:**
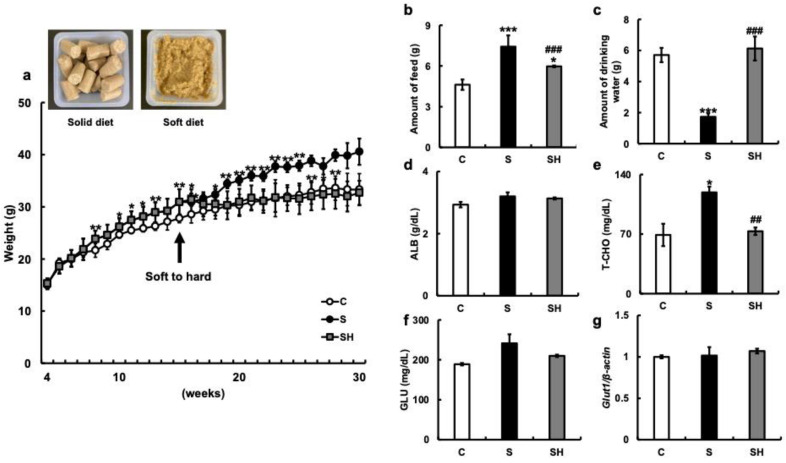
Effects of soft-food rearing on the physiological functions of mice. (**a**) Weight change: The arrow indicates the time point at which certain mice in the soft-fed group resumed a solid diet. Representative images of a typical meal for each type of diet are shown in the upper left corner of the panel. (**b**) The amount of food (g) in the 6th month of rearing. (**c**) The amount of drinking water in the 6th month of rearing. (**d**) Serum albumin levels (g/dL) in the 6th month of rearing. (**e**) Total cholesterol (T-CHO) levels (mg/dL) in the 6th month of rearing. (**f**) Serum glucose levels (mg/dL) in the 6th month of rearing. (**g**) *Glut1* mRNA levels in the hippocampus. One-way ANOVA, Tukey’s post hoc test: * *p* < 0.05, ** *p* < 0.01, *** *p* < 0.001, ^##^ *p* < 0.01, ^###^ *p* < 0.001. * corresponds with C vs. S and C vs. SH comparisons, # corresponds with S vs. SH comparisons. Results are presented as mean ± SE. SE, standard error; ANOVA, analysis of variance; Glut1, glucose transporter 1; C, control group; S, soft-food rearing group; and SH, soft-food and solid diet group.

We first examined the body weight of C57BL/6N mice reared on a solid diet for six months (C group), a soft diet (S group) for six months, and a soft diet that was returned to a solid diet (SH group) after three months (Figure 1a). The soft diet group showed significant weight gain five weeks after the start of the experiment (eight weeks of age) when compared with the solid diet group (*p* = 0.008). Thereafter, group S continued to gain weight. By the end of the experiment, the difference in average weight had increased significantly compared with group C. However, there was no significant difference due to large individual differences (*p* = 0.34). In the SH group, although weight gain was observed during the soft diet feeding period, the body weight began to decrease after switching from the soft to solid diet. The body weight at the end of the experiment was similar to that of the C group (*p* = 0.68).

Food consumption after six months of rearing was significantly higher in the S group (7.42 ± 0.83 g) than in the C group (4.6 ± 0.38 g; *p < 0.001*) (Figure 1b). Conversely, the food intake of the SH group (5.97 ± 0.06 g) was significantly lower than that of the S group (*p* < 0.001). Water intake after six months of rearing was significantly lower in the S group (1.73 ± 0.22 g) than in the C group (5.71 ± 0.46 g) (*p <* 0.001) (Figure 1c) and significantly increased in the SH group (6.12 ± 0.42 g) compared with that in the S group (*p <* 0.001) (Figure 1c). The levels of serum albumin (g/dL) were similar among the three groups (C group = 2.93 ± 0.09 g/dL, S group =3.2 ± 0.13 g/dL, and SH group = 3.13 ± 0.03 g/dL) (Figure 1d). Total cholesterol (T-CHO) levels were higher in the S group than in the C and SH groups (C vs. S, *p* = 0.01; S vs. SH, *p* = 0.003; C vs. SH, *p* = 0.43; C group = 69 ± 13.1 mg/dL, S group = 119 ± 6.75 mg/dL, and SH group = 73.3 ± 4.37 mg/dL; Figure 1e), and serum glucose levels were similar among the three groups (C group = 189 ± 3 mg/dL, S group = 241.5 ± 22.5 mg/dL, and SH group = 210 ± 3 mg/dL; Figure 1f). There was also no significant difference in hippocampal *Glut1* mRNA expression between the three groups (Figure 1g).

### 3.2. Effects of Soft-Food Rearing on Memory and Behavior

**Figure 2:**
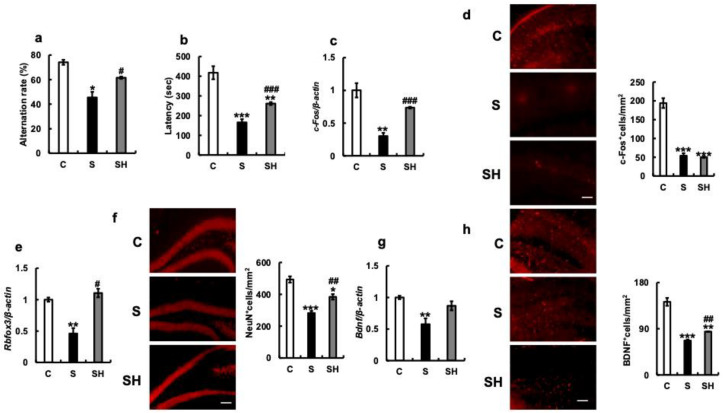
Effects of long-term soft-food rearing on memory and behavior in mice. (**a**) Transformation rate of mice in the maze experiment. (**b**) Latency of mice in the rotarod experiment. (**c**) *c-Fos* mRNA expression in the hippocampus. (**d**) c-Fos immunostaining in the hippocampus. (**e**) *Rbfox3* mRNA expression in the hippocampus. (**f**) NeuN immunostaining in the hippocampus. (**g**) *Bdnf* mRNA expression in the hippocampus. (**h**) BDNF immunostaining in the hippocampus. The target gene expression was normalized to the expression of the housekeeping gene β-actin, and the results are relative to the control for each sample. The number of c-Fos-, NeuN-, and BDNF-positive cells decreased with prolonged soft-food rearing and increased with a return to a solid diet in the SH group. Scale bar, 100 μm. One-way ANOVA, Tukey’s post hoc test: * *p* < 0.05, ** *p* < 0.01, *** *p* < 0.001, ^#^ *p* < 0.05, ^##^ *p* < 0.01, ^###^ *p* < 0.001. * corresponds to C vs. S and C vs. SH comparisons, # corresponds to S vs. SH comparisons. Results are presented as mean ± SE. SE, standard error; ANOVA, analysis of variance; Rbfox3, RNA binding protein fox-1 homolog 3; CA1, hippocampal cornu ammonis; NeuN, neuronal nuclei; BDNF, brain-derived neurotrophic factor; C, control group; S, soft-food rearing group; SH, soft-food and solid diet group.

In the Y-maze test, the S group demonstrated a significantly lower replacement rate than the C group (*p* = 0.04), while the SH group demonstrated a higher recovery rate than the S group (*p* = 0.03, Figure 2a). The effect of soft food feeding on motor learning function was investigated using the rotarod test. The latency in the rotarod test was significantly shorter in the S group than in the C group *(p* = 0.0007, Figure 2b), and slightly longer in the SH group (which switched from the soft to the solid diet) than in the S group (C vs. SH, *p* < 0.001; S vs. SH, *p* < 0.01). These results suggest that balance and motor coordination were impaired in the S group (Figure 2b).

Next, we examined the expression of various genes related to cognitive function and brain activity using extracts from the hippocampus of the mice. The expression of *c-Fos* mRNA in the hippocampus was lower in the S group than in the C group (*p* < 0.01, Figure 2c). Similarly, immunostaining showed that the number of c-Fos-positive cells was decreased in the S group compared to that in the C group (*p* < 0.001, Figure 2d). On the other hand, a significantly higher level of *c-Fos* mRNA expression was observed in the SH group than in the S group (*p* < 0.001, Figure 2c). The mRNA expression of Rbfox3 (NeuN), a neuronal marker, in the hippocampus was significantly decreased in the S group compared to that in the C group (*p* = 0.002, Figure 2e) but increased in the SH group compared to that in the S group (*p* = 0.02). In addition, the number of NeuN-positive cells in the hippocampus decreased in the S group (C vs. S; *p* < 0.001) but increased in the SH group compared with that in the S group (*p* = 0.009, Figure 2f). Compared with the C group, *Bdnf* mRNA levels decreased in the S group (*p =* 0.003) and increased in the SH group (*p* = 0.07; Figure 2g). On the other hand, there was an increase in the BDNF immunostaining signal in the SH group in comparison with that in the S group (*p* = 0.008; Figure 2h).

### 3.3. Twenty-Four-Hour Locomotor Activity

**Figure 3:**
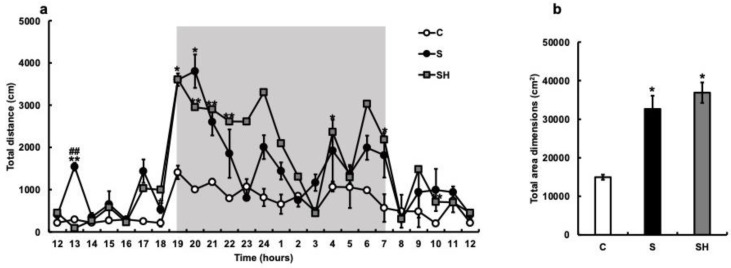
Effects of soft-food rearing on mice behavior. The vertical axis represents the average total distance (cm) per hour. The horizontal axis indicates time. (**a**) Behavioral distance of each mouse for 24 h. (**b**) Behavioral distance, presented as the area, of each mouse for 24 h. All data were obtained at 6 months. One-way ANOVA, Tukey’s post hoc test: * *p* < 0.05, ** *p* < 0.01, ^##^ *p* < 0.01. * corresponds with C vs. S and C vs. SH comparisons, # corresponds with S vs. SH comparisons. Results are presented as mean ± SE. SE, standard error; ANOVA, analysis of variance; C, control group; S, soft-fed rearing group; and SH, soft-food solid diet group.

The amount of locomotion in the breeding cage was measured with an infrared sensor, and the total distance moved per hour was calculated. Compared with the C group, the S group demonstrated an increase in the total distance traveled, with particularly increased activity after 19:00 (Figure 3a). Similarly, nocturnal activity increased in the SH group compared with that in the C group. The total area dimensions (cm^2^) were significantly increased in the S group (C vs. S; *p* = 0.04). Three months after switching from soft to solid food, the SH group also showed an increase in nocturnal activity, with the same trend observed as in the S group (*p* = 0.02; Figure 3b).

### 3.4. ABB toward an Inanimate Object

**Figure 4:**
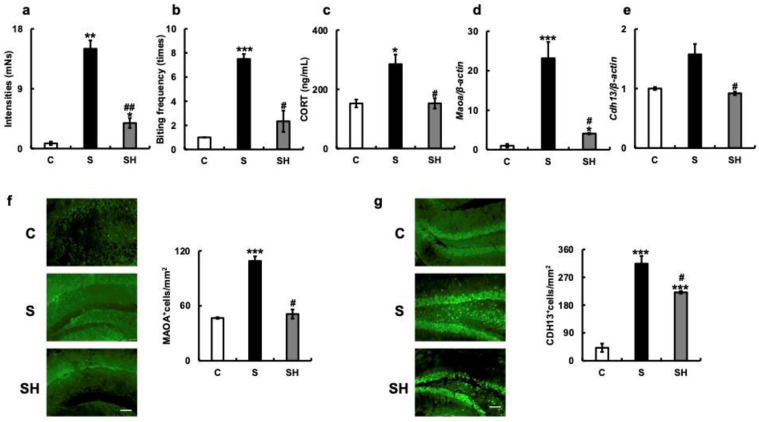
Effects of long-term soft-food rearing on aggression in mice. (**a**) Bite strength (mNs) of mice against inanimate objects. (**b**) Bite frequency (times). (**c**) Serum corticosterone levels (ng/mL), (**d**) *Maoa* mRNA expression in the hippocampus. (**e**) *Cdh13* mRNA expression in the hippocampus. (**f**) MAOA immunostaining in the hippocampus. (**g**) CDH13 immunostaining in the hippocampus. The target gene expression was normalized to the expression of the housekeeping gene β-actin. The results are relative to the control for each sample. The number of MAOA- and CDH13-positive cells increased with soft-diet rearing and decreased with a solid diet in the SH group. All data were obtained at 6 months. Scale bar, 100 μm. One-way ANOVA, Tukey’s post hoc test: * *p* < 0.05, ** *p* < 0.01, *** *p* < 0.001, ^#^ *p* < 0.05, ^##^ *p* < 0.01. * corresponds to C vs. S and C vs. SH comparisons, # corresponds to S vs. SH comparison. Results are presented as mean ± SE. SE, standard error; ANOVA, analysis of variance; MAOA, monoamine oxidase A; CDH13, T-cadherin; C, control group; S, soft-fed rearing group; and SH, soft-food solid diet group.

The intensity of ABB towards inanimate objects was significantly higher in the S group (15.0 ± 1.25 mNs) than in the C group (0.78 ± 0.26 mNs; *p* = 0.001; Figure 4a) and SH group (3.84 ± 0.73 mNs; *p* = 0.005). The bite frequency was higher in the S group (7.5 ± 0.41 times) than in the C group (1 ± 0 times; *p* < 0.001) and SH group (2.33 ± 0.88 times; *p* = 0.023; Figure 4b). Based on the aggression results (Figure 4a,b), serum corticosterone concentrations were examined. Blood corticosterone levels were higher in the S group (285 ± 32.5 ng/mL) than in the C group (152.3 ± 13.3 ng/mL; *p* = 0.02) and SH group (152.67 ± 17.42 ng/mL; *p* = 0.023; Figure 4c).

PCR revealed that the mRNA expression of *Maoa* in the hippocampus was higher in the S group than in the C group (*p* < 0.001) and SH group (*p* = 0.04; Figure 4d). In the S group, the mRNA expression of *Cdh13* in the hippocampus was also higher than in the C group (*p* = 0.08) and significantly higher than in the SH group (*p* = 0.02; Figure 4e). In the S group, the number of MAOA-positive cells in the hippocampus was higher than in the C group (*p* < 0.001) and significantly higher than in the SH group (*p* = 0.02; Figure 4f). Additionally, the number of CDH13-positive cells was significantly higher in the S group than in the C or SH groups (C vs. S, *p* = 0.001; S vs. SH, *p* = 0.02; Figure 4g).

### 3.5. Effects of Soft-Food Rearing on the Expression of Serotonin-Related Molecules

**Figure 5:**
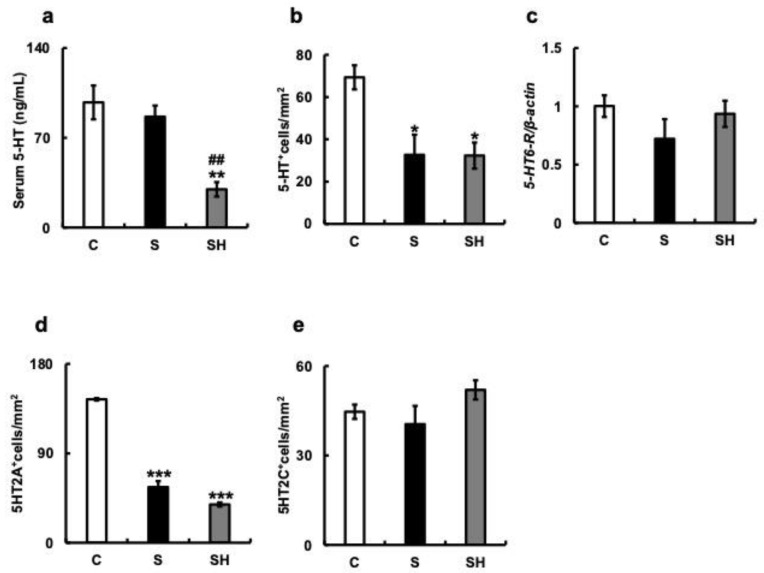
Effects of soft-food rearing on serotonin-related molecules. (**a**) Serum serotonin concentration (ng/mL), (**b**) number of 5-HT-positive cells in the hippocampus, (**c**) *5-HT6-R* mRNA expression level, (**d**) The number of 5-HT2A-positive cells in the hippocampus, and (**e**) The number of 5-HT2C-positive cells in the hippocampus. The target gene expression was normalized to the expression of the housekeeping gene β-actin. The results are relative to the control for each sample. The number of 5-HT-positive cells decreased with soft-diet rearing and remained identical with a solid diet in the SH group. All data were obtained at 6 months. One-way ANOVA, Tukey’s post hoc test: * *p* < 0.05, ** *p* < 0.01, *** *p* < 0.001, ^##^ *p* < 0.01. * corresponds to C vs. S and C vs. SH comparison, # corresponds to S vs. SH comparison. Results are presented as mean ± SE. SE, standard error; ANOVA, analysis of variance; 5-HT, 5-hydroxy-tryptamine; 5-HT6-R, 5-hydroxytryptamine-6 receptor; CA1, hippocampal cornu ammonis; C, control group; S, soft-fed rearing group; and SH, soft-food solid diet group.

We examined the expression of serotonin and serotonin-related molecules in mice with soft-fed rearing based on the results of cognitive decline (Figure 2), increased nocturnal activity (Figure 3), and aggression (Figure 4). Serum 5-HT concentrations in experimental mice were similar between the C group (97.6 ± 13.19 ng/mL) and S group (86.43 ± 8.68 ng/mL) (*p* = 0.57), and significantly lower in the SH group than in both other groups (27.8 ± 5.61 ng/mL) (S vs. SH, *p* = 0.003; C vs. SH, *p* = 0.004; Figure 5a). When 5-HT-expressing cells in the hippocampus were examined after immunostaining, the number was significantly lower in the S and SH groups compared with that in the C group (C vs. S, *p* = 0.03; C vs. SH, *p* = 0.01; Figure 5b). Hippocampal serotonin expression did not differ between the S and SH groups (*p* = 0.97; Figure 5b). Furthermore, when *5-HT6-R* mRNA levels associated with aggression were examined in the hippocampus, there was no difference observed between the three groups (C vs. S, *p* = 0.17; C vs. SH, *p* = 0.67; S vs. SH, *p* = 0.3; Figure 5c).

Additionally, we measured the number of serotonin receptors, including 5-HT2A- and 5-HT2C-positive cells, in the hippocampus by immunostaining. Compared with that in the C group, a decrease in the number of 5-HT2A-positive cells was observed in the S and SH groups (C vs. S, *p* < 0.001; C vs. SH, *p* < 0.001; S vs. SH, *p* = 0.11; Figure 5d). Conversely, the number of 5-HT2C-positive cells did not differ among the three groups (C vs. S, *p* = 0.56; C vs. SH, *p* = 0.14; S vs. SH, *p* = 0.19; Figure 5e).

### 3.6. Effects of Soft-Food Rearing on AMPA Receptor-Related Molecule Expression in the Hippocampus

**Figure 6:**
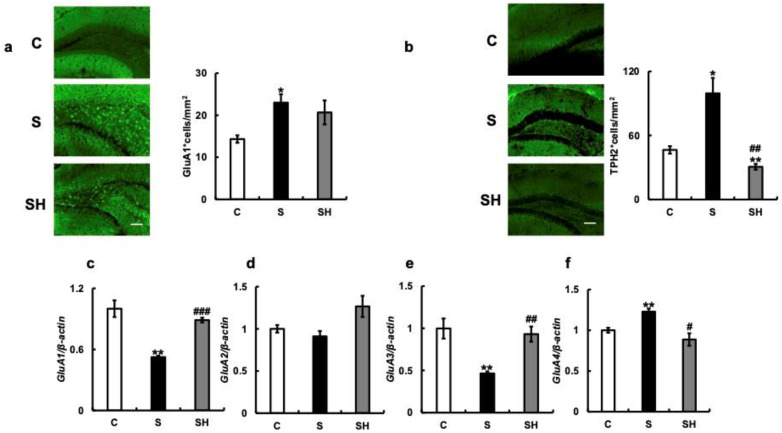
Effects of soft-food rearing on AMPA receptor-related molecules. (**a**) Immunostaining for GluA1 in the hippocampus. (**b**) Immunostaining for TPH2 in the hippocampus. (**c**) *GluA1* mRNA levels in the hippocampus. (**d**) *GluA2* mRNA levels in the hippocampus. (**e**) *GluA3* mRNA levels in the hippocampus. (**f**) *GluA4* mRNA levels in the hippocampus. The target gene expression was normalized to the expression of the housekeeping gene β-actin. The results are relative to the control for each sample. All data were obtained at 6 months. One-way ANOVA, Tukey’s post hoc test: * *p* < 0.05, ** *p* < 0.01, ^#^*p* < 0.05, ^##^ *p* < 0.01, ^###^ *p* < 0.001. * corresponds to C vs. S, C vs. SH comparisons, # corresponds to S vs. SH comparison. Results are presented as mean ± SE. SE, standard error; ANOVA, analysis of variance; GluA1, Glutamate receptor 1, AMPA, α-amino-3-hydroxy-5-methyl-4-isoxazole propionic acid; GluA1, glutamate ionotropic receptor AMPA-type subunit 1; GluA2, glutamate ionotropic receptor AMPA-type subunit 2; GluA3, glutamate ionotropic receptor AMPA-type subunit 3; GluA4, glutamate ionotropic receptor AMPA-type subunit 4; CA1, hippocampal cornu ammonis; C, control group; S, soft-food-fed rearing group; and SH, soft-food solid diet group.

We next examined the expression of GluA1, a subtype of the AMPA receptor (Figure 6a). Protein expression of GluA1 in the hippocampus was higher in the S group (*p* = 0.02) than in the C group (S vs. SH, *p* = 0.6; C vs. SH, *p* = 0.11), while there were no significant differences between the S and SH groups. Next, we examined the expression of TPH2 in relation to AMPA receptor sensitivity. The expression of TPH2 in the hippocampus was higher in the S group than in the C group (*p* = 0.02), and significantly lower in the SH group than in the S group (*p* = 0.009; Figure 6b).

We further examined the expression of mRNA coding for AMPA receptor subunits—GluA1, GluA2, GluA3, and GluA4—in the hippocampus (Figure 6c–f). *GluA1* mRNA expression decreased in the S group compared with that in the C group (*p* = 0.008) and significantly increased in the SH group compared with that in the S group (*p* < 0.001; Figure 6c). *GluA2* mRNA expression did not differ among the three groups (Figure 6d), while *GluA3* mRNA expression decreased in the S group (*p =* 0.002) and increased in the SH group (*p =* 0.001), similar to the change in *GluA1* mRNA expression. Conversely, *GluA4* mRNA increased in the S group compared with that in the C group (*p* = 0.009) and decreased in the SH group compared with that in the S group (*p* = 0.02; Figure 6f).

## 4. Discussion

There are many reports on the relationships between caloric and nutrient intake and health conditions and diseases [38]. However, the relationship between the shape and hardness of dietary staples and systemic health has not been well studied. In this study, we observed differences in cognitive function, motor function, and mood-related behavior between young mice fed a hard diet over an extended period of time and a group fed a soft diet of the same nutritional value. At the same time, changes in molecular expression associated with these changes in the hippocampus were examined. The results demonstrate that the mice fed a soft diet exhibited a decrease in cognitive and motor functions and an increase in nocturnal activity and aggression. Changes in the expression of related molecules were also found. These results suggest a negative health effect of a soft diet on the brain. However, we acknowledge that the behavioral phenotype of mice cannot be fully determined by a single test. Additional tests assessing cognition, aggression, and other relevant behavioral aspects are crucial for a comprehensive evaluation and to confirm the results presented here [39].

Soft-food rearing is known to accelerate aging in mice [13], shorten life span [12], and impair cognitive function [12,23,24]. However, no basic studies have revealed the effects of soft-food rearing on aggression. This study supported the hypothesis that soft-food rearing induces aggressive behavior, despite reduced aggressive behavior, latency, weight gain, and other gene expression changes (except for 5-HT and *Bdnf* in the hippocampus) in the SH group. Although the definition of aggression is straightforward, its origins remain complex and often depend on other, often contradictory factors. Underlying diseases, medications, and hormones are thought to be associated with aggression [40,41]. However, this study is the first to show that food type and diet hardiness are also involved in aggression.

In the present study, the expression of aggression-related genes—such as *Maoa*, *Cdh13*, and *Ampa*—was suppressed in the SH group, which was switched from a soft to a solid diet. However, the expression of hormones that control mental status, such as 5-HT and BDNF, did not improve. Only three months of rearing on a soft diet impaired the expression of these hormones, and three months of subsequent rearing on a solid diet was insufficient to restore them. Considering that the expression of serotonin and BDNF did not improve in the SH group, it is likely that the debilitation of aggression was due to glutamate, not serotonin. Mood and emotion are regulated by monoamines such as noradrenaline and serotonin in the brain [42]. However, they are degraded by MAOA, and it is thought that increased MAOA levels and activity are involved in increased anxiety and other emotions, as well as aggression [43]. The *Maoa* gene is also known for its involvement in aggression [44] and encodes an enzyme important for the degradation of serotonin and catecholamines [45]. Congenital MAOA deficiency and constantly active MAOA variants are associated with the risk of violence [42]. Recently, MAOA has been associated with pathological internet use (PIU) [46], with high *Maoa* mRNA expression and high MAOA activity reported in patients with PIU. Notably, strong correlations were observed between PIU and depression. Animals exposed to chronic stress and patients with depression exhibit elevated MAOA activity in the brain [35,47,48]. Moreover, the increase in *Maoa* mRNA was associated with changes in monoamine neurotransmitters. Considering the prevalence of aggression in certain patients with major depression [49], it is possible that prolonged soft-food rearing may have caused depression and increased aggression in our study.

CDH13, which is involved in intercellular adhesion, activates small GTPases and the β-catenin/Wnt pathway and plays an important role in cytoskeletal reorganization. The molecule has also been associated with attention-deficit/hyperactivity disorder, autism, schizophrenia, substance abuse, and bipolar disorder. Additionally, the CDH13 genotype has been reported to be associated with severe violent crime [50]. In this study, the increased aggression and nocturnal activity observed in the S group was attributed to the increased expression of the *Maoa* and *Cdh13* genes in the hippocampus of these mice (Figure 4d,e). The 5-HT2A receptor (5-HT2AR) enhances glutamatergic synaptic transmission [51] and has been implicated in major depression in peripheral blood mononuclear cells. Additionally, *5-HT2AR* mRNA levels in brain tissue of affected individuals have been reported to be significantly elevated compared with those in healthy subjects [52,53,54].

The increased expression of *Maoa*, coupled with the decreased number of serotonin-positive cells and decreased 5-HT2A receptor expression (Figure 5b,d), may have contributed to the increased anxiety and aggression. TPH2 is a potential target for psychiatric disease treatment due to its critical role in 5-HT neurotransmission [55] as a rate-limiting enzyme [56]. TPH2 expression may cause aggression [57,58], as increased impulsivity, excessive aggression, and other behavioral traits are associated with mutations in the *Tph2* gene, a key enzyme in brain serotonin synthesis [56,57,58]. In the present study, more *Tph2*-positive cells were found in the hippocampus of soft-fed mice than in solid-fed mice (Figure 6b), which may also be related to increased aggression. In the SH group, a decrease in aggression was observed along with a decrease in *Maoa* and *Cdh13* gene expression. This suggests that a solid diet regulates *Maoa*, *Cdh13*, and TPH2 expression, which may be responsible for the decrease in aggression. Results concerning the C group suggest that a solid diet may have reduced aggression. However, the change to a solid diet did not decrease the increase in nocturnal activity, suggesting that the expression of those molecules did not affect this change in activity. The 5-HT system plays an important role in regulating aggression [26]. Stress enhances 5-HT deficiency in the brain, thus decreasing 5-HT in mice neurons [59] and inducing aggression [60]. Chronic and acute stress have thus been associated with aggression and violent behavior [42,61].

In the present study, soft-fed mice with elevated serum corticosterone levels also exhibited a decrease in 5-HT-positive cells (Figure 5b). At the same time, aggression increased in those mice (Figure 4a,b). Conversely, serum corticosterone levels decreased in the C and SH groups, suggesting that a solid diet may reduce stress levels (Figure 4c). In soft-fed mice, decreased levels of *c-Fos*, *Bdnf*, and *NeuN* mRNA were observed (Figure 2), and reports have shown that these changes are associated with cognitive decline [23]. BDNF is one of the neurotrophic factors reported to be associated with depression, Alzheimer’s disease, and cognitive functions (such as memory and learning) and is also reported to be decreased in individuals with dementia [62]. Stress reduces *Bdnf* mRNA expression in the hippocampus of animals [63]. Supporting this, decreased *BDNF* levels in the serum have been observed in patients with mood disorders under excessive stress and depression [64,65]. The BDNF and serotonin systems interact with each other to regulate the development and plasticity of neural circuits involved in mood disorders, including antidepressant responses [66]. By contrast, heterozygous mice with the *Bdnf* gene exhibit abnormal behaviors, such as overeating, anxiety, and aggression [67,68]. Nonetheless, *Bdnf* expression in the hippocampus and nighttime behavior increased. These were worse in the SH group than in both the S and C groups. Inadequate masticatory activity during development reduced the BDNF protein levels in the hippocampus, thereby affecting synaptic plasticity in the region [69]. In addition, reduced masticatory activity due to a soft diet increases stress levels, which in turn decreases BDNF levels. This may be related to increased aggression, as well as decreased cognitive function.

The *Ampa* receptor is an important ion channel-type receptor responsible for excitatory neurotransmission in the central nervous system. This receptor is composed of a tetramer of four subunits (GluA1-4), and differences in subunit composition are thought to reflect physiological properties [70]. Mice deficient in the *Ampa* receptor subtypes GluA1 and GluA3 exhibit decreased and increased aggression, respectively [71]. In a study of ultrasound irradiation-induced aggression [72], the irradiated group revealed decreased hippocampal *GluA1* mRNA, increased *GluA2* mRNA, decreased *GluA3* mRNA, and unchanged *GluA4* mRNA expression, compared with controls. Our findings support their data, and although the methods of evaluation are different, their results approximate those of the present study. Thus, the results of this study suggest that soft-food rearing alters the subunit composition of AMPA receptors.

This study has several limitations. Aggression-related neurobiological factors involve the action of the prefrontal cortex and its interconnection with midbrain structures, such as the amygdala, hypothalamus, and periaqueductal gray, which are involved in the acute threat response system [73]. The hippocampus, while densely projecting to multiple brain regions, may also have direct involvement in the control of impulsive/aggressive behavior through its connection with the amygdala [74]. Furthermore, these brain regions play a role in regulating the hypothalamus-pituitary-adrenal stress response system [75]. As our study initially focused on investigating the relationship between soft-food rearing and cognitive function, we chose to examine the molecular dynamics within the hippocampus. However, in order to elucidate the molecular mechanisms underlying increased aggression, it would be necessary to analyze other brain regions such as the amygdala, prefrontal cortex, and midbrain. To assess the molecular changes associated with the observed differences in cognitive function, motor function, and mood-related behavior, Western blot analysis was not performed in this study. Instead, we utilized PCR and immunostaining techniques to examine the expression of relevant molecules. Given that this study has identified candidate molecules exhibiting fluctuations, we intend to further analyze these other brain regions in future research based on the findings of this study.

In conclusion, the results of this study indicate that long-term rearing of young mice on a soft diet causes weight gain, cognitive decline, and loss of balance and motor coordination—as previously reported—as well as anxiety and mood regulation-related behavioral changes (such as increased nocturnal activity and aggression). Conversely, these changes were suppressed or ameliorated by feeding solid food or switching to a solid diet. Although the results of this study were obtained in a murine model, they are important findings that reaffirm the importance of food firmness and chewing for the healthy development of brain function. We sincerely hope that further analysis in humans will be conducted in the future.

## Figures and Tables

**Table 1:** Sequences of primers used for real-time PCR.

Name	Forward	Reverse
Mouse *Glut1*	TCAACACGGCCTTCACTG	CACGATGCTCAGATAGGACATC
Mouse *Fos* (c-Fos)	GGGGACAGCCTTTCCTACTA	CTGTCACCGTGGGGATAAAG
Mouse *Rbfox3* (NeuN)	CACCACTCTCTTGTCCGTTTGC	GGCTGAGCATATCTGTAAGCTGC
Mouse *Bdnf*	TGCAGGGGCATAGACAAAAGG	CTTATGAATCGCCAGCCAATTCTC
Mouse *Maoa*	TCAATGTAGCCACTCCACTGT	TTGGGGATAAAGTGAAGCTGA
Mouse *Cdh13*	GGCAATTGACAGTGGCAACC	TGCAGGAGCACACTTGTACC
Mouse *5-HT6-R*	GCATAGCTCAGGCCGTATGT	CACCACTGTGAGAGGTCCAC
Mouse *GluA1*	GGACAACTCAAGCGTCCAGA	GTCGGTAGGAATAGCCCACG
Mouse *GluA2*	GCGTGGAAATAGAAAGGGCC	ACTCCAGTACCCAATCTTCCG
Mouse *GluA3*	ACCATCAGCATAGGTGGACTT	ACGTGGTAGTTCAAATGGAAGG
Mouse *GluA4*	GGCCAGGGAATTGACATGGA	CCTTTCGAGGTCCTGTGCTT
Mouse *β-actin*	TGTGATGGTGGGAATGGGTCAGAA	TGTGGTGCCAGATCTTCTCCATGT

*Glut1*, glucose transporter 1; *Rbfox3*, RNA binding protein fox-1 homolog 3; NeuN, neuronal nuclei; *Bdnf*, brain-derived neurotrophic factor; *Maoa*, monoamine oxidase A; *Cdh13*, T-cadherin; *5-HT6-R*, 5-hydroxytryptamine-6 receptor; *β-actin*, beta actin; *GluA1*, glutamate ionotropic receptor AMPA-type subunit 1; *GluA2*, glutamate ionotropic receptor AMPA-type subunit 2; *GluA3*, glutamate ionotropic receptor AMPA-type subunit 3; and *GluA4*, glutamate ionotropic receptor AMPA-type subunit 4.

## Data Availability

The source data are available as source data files. All other data are available from the corresponding authors upon reasonable request.

## Supplementary Materials

The following supporting information can be downloaded at: https://www.mdpi.com/2072-6643/15/10/2397/s1, Figure S1: Representative images of the standard pellets and the powder provided as hard and soft diet, respectively, to the experimental animals in each group.

## References

[1] Healthy Diet. https://www.who.int/news-room/fact-sheets/detail/healthy-diet.

[2] Galiniak S., Aebisher D., Bartusik-Aebisher D. (2019). Health benefits of resveratrol administration. Acta Biochim. Pol..

[3] Sunkara A., Raizner A. (2019). Supplemental vitamins and minerals for cardiovascular disease prevention and treatment. Methodist. DeBakey Cardiovasc. J..

[4] Forde C.G., Bolhuis D. (2022). Interrelations between food form, texture, and matrix influence energy intake and metabolic responses. Curr. Nutr. Rep..

[5] van der Bilt A., Engelen L., Pereira L.J., van der Glas H.W., Abbink J.H. (2006). Oral physiology and mastication. Physiol. Behav..

[6] Kubo K.Y., Iinuma M., Azuma K. (2015). Mastication as a stress-coping behavior. BioMed Res. Int..

[7] Hori K., Uehara F., Yamaga Y., Yoshimura S., Okawa J., Tanimura M., Ono T. (2021). Reliability of a novel wearable device to measure chewing frequency. J. Prosthodont. Res..

[8] Hamada Y., Hayashi N. (2021). Chewing increases postprandial diet-induced thermogenesis. Sci. Rep..

[9] Onyper S.V., Carr T.L., Farrar J.S., Floyd B.R. (2011). Cognitive advantages of chewing gum. Now you see them, now you don’t. Appetite.

[10] Pedersen A.M.L., Sørensen C.E., Proctor G.B., Carpenter G.H., Ekström J. (2018). Salivary secretion in health and disease. J. Oral Rehabil..

[11] Tada A., Miura H. (2018). Association of mastication and factors affecting masticatory function with obesity in adults: A systematic review. BMC Oral Health.

[12] Yamamoto T., Hirayama A. (2001). Effects of soft-diet feeding on synaptic density in the hippocampus and parietal cortex of senescence-accelerated mice. Brain Res..

[13] Tsuchiya M., Niijima-Yaoita F., Yoneda H., Chiba K., Tsuchiya S., Hagiwara Y., Sasaki K., Sugawara S., Endo Y., Tan-No K. (2014). Long-term feeding on powdered food causes hyperglycemia and signs of systemic illness in mice. Life Sci..

[14] Yoshino F., Yoshida A., Hori N., Ono Y., Kimoto K., Onozuka M., Lee M.C. (2012). Soft-food diet induces oxidative stress in the rat brain. Neurosci. Lett..

[15] Ono Y., Yamamoto T., Kubo K.Y., Onozuka M. (2010). Occlusion and brain function: Mastication as a prevention of cognitive dysfunction. J. Oral Rehabil..

[16] Mori D., Katayama T., Miyake H., Fujiwara S., Kubo K.-Y. (2013). Occlusal disharmony leads to learning deficits associated with decreased cellular proliferation in the hippocampal dentate gyrus of SAMP8 mice. Neurosci. Lett..

[17] Furukawa M., Tada H., Wang J., Yamada M., Kurosawa M., Satoh A., Ogiso N., Shikama Y., Matsushita K. (2022). Molar loss induces hypothalamic and hippocampal astrogliosis in aged mice. Sci. Rep..

[18] Teixeira F.B., Pereira Fernandes L.d.M., Noronha P.A., dos Santos M.A., Gomes-Leal W., Ferraz Maia C.d.S., Lima R.R. (2014). Masticatory deficiency as a risk factor for cognitive dysfunction. Int. J. Med. Sci..

[19] Frota de Almeida M.N., de Siqueira Mendes F.d.C.C., Gurgel Felício A.P., Falsoni M., Ferreira de Andrade M.L., Bento-Torres J., da Costa Vasconcelos P.F., Perry V.H., Picanço-Diniz C.W., Kronka Sosthenes M.C. (2012). Spatial memory decline after masticatory deprivation and aging is associated with altered laminar distribution of CA1 astrocytes. BMC Neurosci..

[20] Aguayo-Mendoza M., Santagiuliana M., Ong X., Piqueras-Fiszman B., Scholten E., Stieger M. (2020). How addition of peach gel particles to yogurt affects oral behavior, sensory perception and liking of consumers differing in age. Food Res. Int..

[21] Wallace R., Lombardi K., Backer C.D., Costello L., Devine A. (2020). Sharing is caring: A study of food-sharing practices in Australian early childhood education and care services. Nutrients.

[22] Okihara H., Ito J.-I., Kokai S., Ishida T., Hiranuma M., Kato C., Yabushita T., Ishida K., Ono T., Michikawa M. (2014). Liquid diet induces memory impairment accompanied by a decreased number of hippocampal neurons in mice. J. Neurosci. Res..

[23] Fukushima-Nakayama Y., Ono T., Hayashi M., Inoue M., Wake H., Ono T., Nakashima T. (2017). Reduced mastication impairs memory function. J. Dent. Res..

[24] Kubo K.-Y., Ichihashi Y., Kurata C., Iinuma M., Mori D., Katayama T., Miyake H., Fujiwara S., Tamura Y. (2010). Masticatory function and cognitive function. Okajimas Folia Anat. Jpn..

[25] Connor D.F., Newcorn J.H., Saylor K.E., Amann B.H., Scahill L., Robb A.S., Jensen P.S., Vitiello B., Findling R.L., Buitelaar J.K. (2019). Maladaptive aggression: With a focus on impulsive aggression in children and adolescents. J. Child. Adolesc. Psychopharmacol..

[26] Raaijmakers M.A., Posthumus J.A., van Hout B.A., van Engeland H., Matthys W. (2011). Cross-sectional study into the costs and impact on family functioning of 4-year-old children with aggressive behavior. Prev. Sci..

[27] Laninga-Wijnen L., Harakeh Z., Steglich C., Dijkstra J.K., Veenstra R., Vollebergh W. (2017). The norms of popular peers moderate friendship dynamics of adolescent aggression. Child. Dev..

[28] Taubner S., Zimmermann L., Ramberg A., Schröder P. (2016). Mentalization mediates the relationship between early maltreatment and potential for violence in adolescence. Psychopathology.

[29] Khayyatzadeh S.S., Mehramiz M., Mirmousavi S.J., Mazidi M., Ziaee A., Kazemi-Bajestani S.M.R., Ferns G.A., Moharreri F., Ghayour-Mobarhan M. (2018). Adherence to a dash-style diet in relation to depression and aggression in adolescent girls. Psychiatry Res..

[30] Kalantari N., Doaei S., Gordali M., Rahimzadeh G., Gholamalizadeh M. (2016). The association between dairy intake, simple sugars and body mass index with expression and extent of anger in female students. Iran. J. Psychiatry.

[31] O’Neill J., Brock C., Olesen A.E., Andresen T., Nilsson M., Dickenson A.H. (2012). Unravelling the mystery of capsaicin: A tool to understand and treat pain. Pharmacol. Rev..

[32] Wahl F., Allix M., Plotkine M., Boulu R.G. (1992). Neurological and behavioral outcomes of focal cerebral ischemia in rats. Stroke.

[33] Kuchiiwa T., Kuchiiwa S. (2016). Evaluation of aggressiveness of female mice using a semi-automated apparatus for measurement of aggressive biting behavior toward an inanimate object. J. Neurosci. Methods.

[34] Yamasaki N., Maekawa M., Kobayashi K., Kajii Y., Maeda J., Soma M., Takao K., Tanda K., Ohira K., Toyama K. (2008). Alpha-CaMKII deficiency causes immature dentate gyrus, a novel candidate endophenotype of psychiatric disorders. Mol. Brain.

[35] Chiuccariello L., Houle S., Miler L., Cooke R.G., Rusjan P.M., Rajkowska G., Levitan R.D., Kish S.J., Kolla N.J., Ou X. (2014). Elevated monoamine oxidase a binding during major depressive episodes is associated with greater severity and reversed neurovegetative symptoms. Neuropsychopharmacology.

[36] Schmittgen T.D., Livak K.J. (2008). Analyzing real-time PCR data by the comparative C(T) method. Nat. Protoc..

[37] Kanda Y. (2013). Investigation of the freely available easy-to-use software “EZR” for medical statistics. Bone Marrow Transplant..

[38] Napoleão A., Fernandes L., Miranda C., Marum A.P. (2021). Effects of calorie restriction on health span and insulin resistance: Classic calorie restriction diet vs. ketosis-inducing diet. Nutrients.

[39] Karl T., Pabst R., von Hörsten S. (2003). Behavioral phenotyping of mice in pharmacological and toxicological research. Exp. Toxicol. Pathol..

[40] Soreff S.M., Gupta V., Wadhwa R., Arif H. (2023). Aggression. StatPearls.

[41] Hamon M., Blier P. (2013). Monoamine neurocircuitry in depression and strategies for new treatments. Prog. Neuropsychopharmacol. Biol. Psychiatry.

[42] Gescher D.M., Kahl K.G., Hillemacher T., Frieling H., Kuhn J., Frodl T. (2018). Epigenetics in personality disorders: Today’s insights. Front. Psychiatry.

[43] Yanowitch R., Coccaro E.F. (2011). The neurochemistry of human aggression. Adv. Genet..

[44] Coccaro E.F., Fanning J.R., Phan K.L., Lee R. (2015). Serotonin and impulsive aggression. CNS Spectr..

[45] Kolla N.J., Bortolato M. (2020). The role of monoamine oxidase A in the neurobiology of aggressive, antisocial, and violent behavior: A tale of mice and men. Prog. Neurobiol..

[46] Qiu M., Zhang C., Dai Y., Zhang L., Wang Y., Peng W., Chen Y., Wen C., Li H., Zhu T. (2021). MRNA levels of MAOA and 5-HT 2 A receptor in patients with pathological internet use: Correlations with comorbid symptoms. Front. Psychiatry.

[47] Grunewald M., Johnson S., Lu D., Wang Z., Lomberk G., Albert P.R., Stockmeier C.A., Meyer J.H., Urrutia R., Miczek K.A. (2012). Mechanistic role for a novel glucocorticoid-KLF11 (TIEG2) protein pathway in stress-induced monoamine oxidase A expression. J. Biol. Chem..

[48] Liu Q., Cole D.A. (2021). Aggressive outbursts among adults with major depressive disorder: Results from the collaborative psychiatric epidemiological surveys. J. Psychiatr. Res..

[49] Tiihonen J., Rautiainen M.R., Ollila H.M., Repo-Tiihonen E., Virkkunen M., Palotie A., Pietiläinen O., Kristiansson K., Joukamaa M., Lauerma H. (2015). Genetic background of extreme violent behavior. Mol. Psychiatry.

[50] Ciranna L. (2006). Serotonin as a modulator of glutamate- and GABA-mediated neurotransmission: Implications in physiological functions and in pathology. Curr. Neuropharmacol..

[51] Lin X., Huang L., Huang H., Ke Z., Chen Y. (2022). Disturbed relationship between glucocorticoid receptor and 5-HT1AR/5-HT2AR in ADHD rats: A correlation study. Front. Neurosci..

[52] Zhang G., Stackman R.W. (2015). The role of serotonin 5-HT2A receptors in memory and cognition. Front. Pharmacol..

[53] Muguruza C., Miranda-Azpiazu P., Díez-Alarcia R., Morentin B., González-Maeso J., Callado L.F., Meana J.J. (2014). Evaluation of 5-HT2A and MGlu2/3 receptors in postmortem prefrontal cortex of subjects with major depressive disorder: Effect of antidepressant treatment. Neuropharmacology.

[54] Pritchard A.L., Harris J., Pritchard C.W., Coates J., Haque S., Holder R., Bentham P., Lendon C.L. (2008). Role of 5HT2A and 5HT2C polymorphisms in behavioural and psychological symptoms of Alzheimer’s disease. Neurobiol. Aging.

[55] Porter R.J., Gallagher P., Watson S., Young A.H. (2004). Corticosteroid-serotonin interactions in depression: A review of the human evidence. Psychopharmacology.

[56] Gorlova A., Ortega G., Waider J., Bazhenova N., Veniaminova E., Proshin A., Kalueff A.V., Anthony D.C., Lesch K.-P., Strekalova T. (2020). Stress-induced aggression in heterozygous TPH2 mutant mice is associated with alterations in serotonin turnover and expression of 5-HT6 and AMPA subunit 2A receptors. J. Affect. Disord..

[57] Lesch K.-P., Araragi N., Waider J., van den Hove D., Gutknecht L. (2012). Targeting brain serotonin synthesis: Insights into neurodevelopmental disorders with long-term outcomes related to negative emotionality, aggression and antisocial behaviour. Philos. Trans. R. Soc. Lond. B Biol. Sci..

[58] Strekalova T., Svirin E., Waider J., Gorlova A., Cespuglio R., Kalueff A., Pomytkin I., Schmitt-Boehrer A.G., Lesch K.-P., Anthony D.C. (2021). Altered behaviour, dopamine and norepinephrine regulation in stressed mice heterozygous in TPH2 gene. Prog. Neuropsychopharmacol. Biol. Psychiatry.

[59] Sachs B.D., Jacobsen J.P.R., Thomas T.L., Siesser W.B., Roberts W.L., Caron M.G. (2013). The effects of congenital brain serotonin deficiency on responses to chronic fluoxetine. Transl. Psychiatry.

[60] Vogel S., Schwabe L. (2019). Stress, aggression, and the balance of approach and avoidance. Psychoneuroendocrinology.

[61] Conway C.C., Keenan-Miller D., Hammen C., Lind P.A., Najman J.M., Brennan P.A. (2012). Coaction of stress and serotonin transporter genotype in predicting aggression at the transition to adulthood. J. Clin. Child. Adolesc. Psychol..

[62] Amidfar M., de Oliveira J., Kucharska E., Budni J., Kim Y.-K. (2020). The role of CREB and BDNF in neurobiology and treatment of Alzheimer’s disease. Life Sci..

[63] Duman R.S., Monteggia L.M. (2006). A neurotrophic model for stress-related mood disorders. Biol. Psychiatry.

[64] Shimizu E., Hashimoto K., Okamura N., Koike K., Komatsu N., Kumakiri C., Nakazato M., Watanabe H., Shinoda N., Okada S. (2003). Alterations of serum levels of brain-derived neurotrophic factor (BDNF) in depressed patients with or without antidepressants. Biol. Psychiatry.

[65] Karege F., Vaudan G., Schwald M., Perroud N., La Harpe R. (2005). Neurotrophin levels in postmortem brains of suicide victims and the effects of antemortem diagnosis and psychotropic drugs. Brain Res. Mol. Brain Res..

[66] Martinowich K., Lu B. (2008). Interaction between BDNF and serotonin: Role in mood disorders. Neuropsychopharmacology.

[67] Kernie S.G. (2000). BDNF regulates eating behavior and locomotor activity in mice. EMBO J..

[68] Lyons W.E., Mamounas L.A., Ricaurte G.A., Coppola V., Reid S.W., Bora S.H., Wihler C., Koliatsos V.E., Tessarollo L. (1999). Brain-derived neurotrophic factor-deficient mice develop aggressiveness and hyperphagia in conjunction with brain serotonergic abnormalities. Proc. Natl. Acad. Sci. USA.

[69] Yamamoto T., Hirayama A., Hosoe N., Furube M., Hirano S. (2009). Soft-diet feeding inhibits adult neurogenesis in hippocampus of mice. Bull. Tokyo Dent. Coll..

[70] Jacobi E., von Engelhardt J. (2017). Diversity in AMPA receptor complexes in the brain. Curr. Opin. Neurobiol..

[71] Adamczyk A., Mejias R., Takamiya K., Yocum J., Krasnova I.N., Calderon J., Cadet J.L., Huganir R.L., Pletnikov M.V., Wang T. (2012). GluA3-deficiency in mice is associated with increased social and aggressive behavior and elevated dopamine in striatum. Behav. Brain Res..

[72] Gorlova A., Pavlov D., Anthony D.C., Ponomarev E.D., Sambon M., Proshin A., Shafarevich I., Babaevskaya D., Lesch K.-P., Bettendorff L. (2019). Thiamine and benfotiamine counteract ultrasound-induced aggression, normalize AMPA receptor expression and plasticity markers, and reduce oxidative stress in mice. Neuropharmacology.

[73] Bartholow B.D. (2018). The aggressive brain: Insights from neuroscience. Curr. Opin. Psychol..

[74] Mitchell M.R., Potenza M.N. (2014). Recent insights into the neurobiology of impulsivity. Curr. Addict. Rep..

[75] Walker S.E., Papilloud A., Huzard D., Sandi C. (2018). The link between aberrant hypothalamic–pituitary–adrenal axis activity during development and the emergence of aggression—Animal studies. Neurosci. Biobehav. Rev..

