# Correction: Furukawa et al. Long-Term Soft-Food Rearing in Young Mice Alters Brain Function and Mood-Related Behavior. *Nutrients* 2023, *15*, 2397

**DOI:** 10.3390/nu16040480

**Published:** 2024-02-07

**Authors:** Masae Furukawa, Hirobumi Tada, Resmi Raju, Jingshu Wang, Haruna Yokoi, Mitsuyoshi Yamada, Yosuke Shikama, Kenji Matsushita

**Affiliations:** 1 Department of Oral Disease Research, Geroscience Research Center, National Center for Geriatrics and Gerontology, Obu 474-8511, Japan; karuvachattu@gmail.com (R.R.); wjsh@ncgg.go.jp (J.W.); haruna.yokoi@ncgg.go.jp (H.Y.); mitsuyos@dpc.agu.ac.jp (M.Y.); shikama@ncgg.go.jp (Y.S.); 2 Department of Nutrition, Faculty of Wellness, Shigakkan University, Obu 474-8651, Japan; tada@sgk.ac.jp; 3 Department of Integrative Physiology, Geroscience Research Center, National Center for Geriatrics and Gerontology, Obu 474-8511, Japan; 4 Department of Operative Dentistry, School of Dentistry, Aichi Gakuin University, Nagoya 464-8651, Japan

## Text Correction

In the original publication [1], we would like to delete this paragraph from the Discussion section: “(Patent No. 4858996, Stimulus Response Measurement System and Stimulus Method Measurement Method). This technology focuses on the property of mice with stress disorders or depressive symptoms that dislike and attack objects that come into physical contact with their bodies. While normal animals do not react well to being poked with a stick, mentally ill animals cannot tolerate stick contact at all and try to eliminate it with violent biting. This aggressive behavior that occurs in psychotic animals is referred to as object-aggressive behavior. The more severe the psychiatric symptoms of the animal, the more aggressive the animal is. Therefore, by measuring the force with which an animal bites a stick, we can evaluate the severity of the animal’s psychiatric symptoms. Since this is a mechanical measurement, there is little room for subjectivity, and it is difficult for results to differ between researchers. The technology we developed was transferred to Muromachi Machinery Co.” The paragraph that was removed contained an extensive description of the aggression measurement device we utilized, which may inadvertently imply that we developed this device.

The corrected text in the Discussion section is as follows:

“Considering that the expression of serotonin and BDNF did not improve in the SH group, it is likely that the debilitation of aggression was due to glutamate, not serotonin. Mood and emotion are regulated by monoamines such as noradrenaline and serotonin in the brain [42]”. 

The authors apologize for any inconvenience caused and state that the scientific conclusions are unaffected. This correction was approved by the Academic Editor. The original publication has also been updated.
