# Supplementary material for: Long-Term Soft-Food Rearing in Young Mice Alters Brain Function and Mood-Related Behavior
Source: Nutrients. 2023 May 20;15(10):2397. doi: 10.3390/nu15102397 (PMC10222696; doi:10.3390/nu15102397)
Supplement: Supplementary file 1 [file nutrients-15-02397-s001.zip › nutrients-2379357-supplementary.pdf]

nutrients

Supplementary Materials

Long-term Soft-food Rearing in Young Mice Alters Brain Function and Mood-related Behavior

Masae Furukawa [1,*], Hirobumi Tada [2,3], Resmi Raju [1], Jingshu Wang [1], Haruna Yokoi [1], Mitsuyoshi Yamada [1,4], Yosuke Shikama [1] and Kenji Matsushita [1,*]

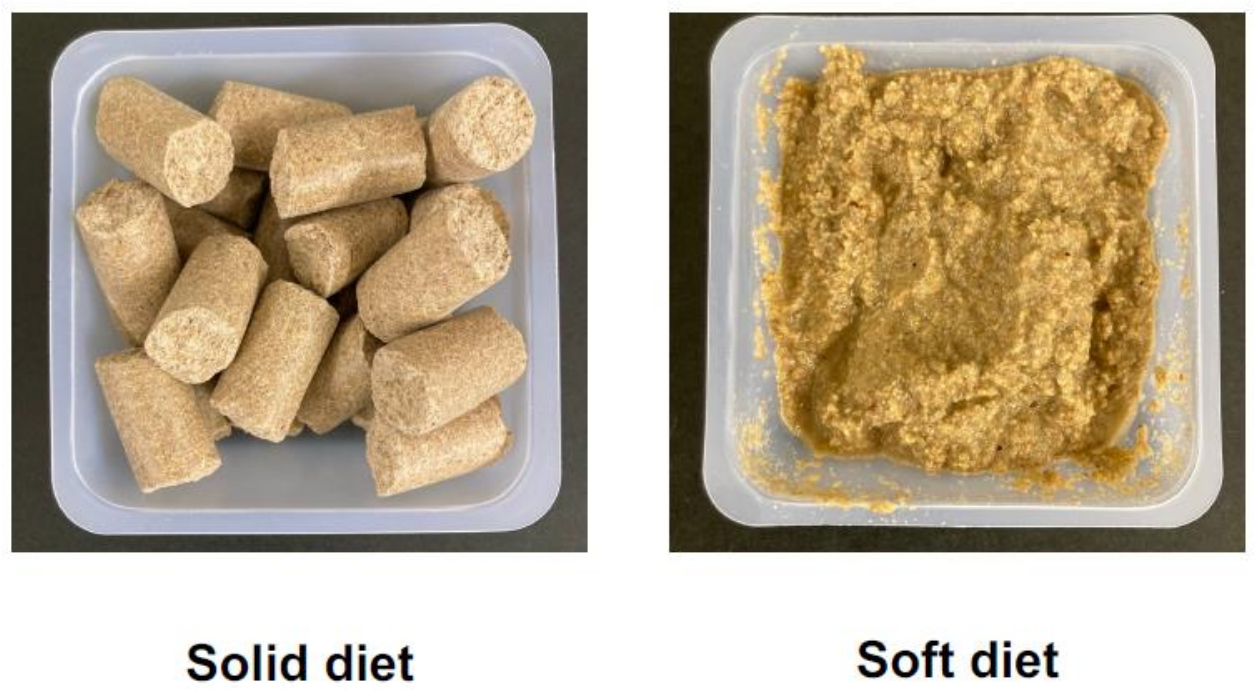

Figure S1. Representative images of the standard pellets and the powder provided as hard and soft diet, respectively, to the experimental animals in each group.
